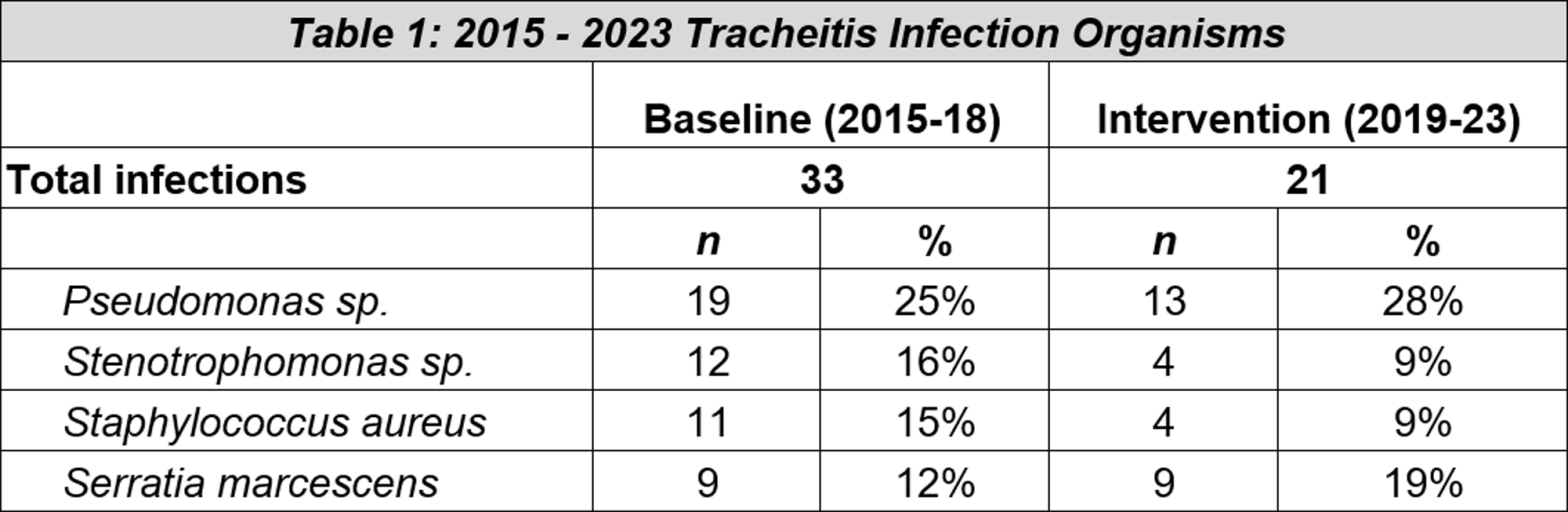# The Difference We Make at Home: Impact of Infection Prevention and Control in Pediatric Homecare Tracheitis Reduction

**DOI:** 10.1017/ash.2024.294

**Published:** 2024-09-16

**Authors:** Sara Griffith, Tom Javens, Sarah King, Christine Zepfel, Carrie Spies, Luke Vohsing, Jessica Parsons, Matthew Washam

**Affiliations:** Nationwide Children’s Hospital

## Abstract

**Background:** Quality improvement (QI) efforts within Infection prevention and control (IP&C) programs to reduce risk of device-related infections in the acute care setting are well described. However, less focus has been placed on continued prevention in the homecare setting. This QI project illustrates the benefits of IP&C involvement in reducing tracheitis in pediatric homecare patients. **Methods:** The homecare multidisciplinary IP&C team implemented a series of QI initiatives aimed at reducing incidence of tracheitis beginning in 2016. Initial interventions included increasing oral care frequency to every four hours, inpatient training for new tracheostomy patients and families before discharge, and an optional inpatient simulation training resource to provide hands-on practice. Enhanced educational interventions included caregiver learning modules and competencies completed with their primary nurse in the home every ninety days and following a tracheitis infection. Practice changes and education efforts were further sustained with the creation and distribution of laminated tracheostomy care teaching sheets to patient homes. Quarterly tracheitis infection rates were tracked using a U-chart. Organism distribution in tracheitis cases were compared across the baseline (2015-2018) and post-intervention periods (2019-2023) using the Chi square test. Analyses were performed using Stata Statistical Software: Release 18 (College Station, TX: StataCorp, LLC) with two-tailed alpha level of 0.05. **Results:** Quarterly tracheitis infection rates from 2015 through 2023 are displayed in the Figure. Notably, the baseline period, established Q1 2015 through Q4 2017, revealed a consistent rate of 1.08 tracheitis infections per 1000 tracheostomy days. During this initial phase, changes in oral care frequency and enhanced educational resources were implemented to decrease rates. Following these interventions, a significant shift was observed in Q1 2019, with the new baseline rate drastically reduced to 0.32 infections per 1000 tracheostomy days. This denotes a remarkable 70% improvement from the prior average infection rate which has been sustained through Q4 2023 with the laminated teaching sheets. The most frequently identified organisms across both time periods are displayed in the Table. Pathogen distribution was similar following QI interventions (p = 0.50). **Conclusions:** Tracheitis infections were reduced by 70% through implementation of multidisciplinary homecare IP&C QI efforts. IP&C programs are integral to pediatric homecare.

**Figure:**
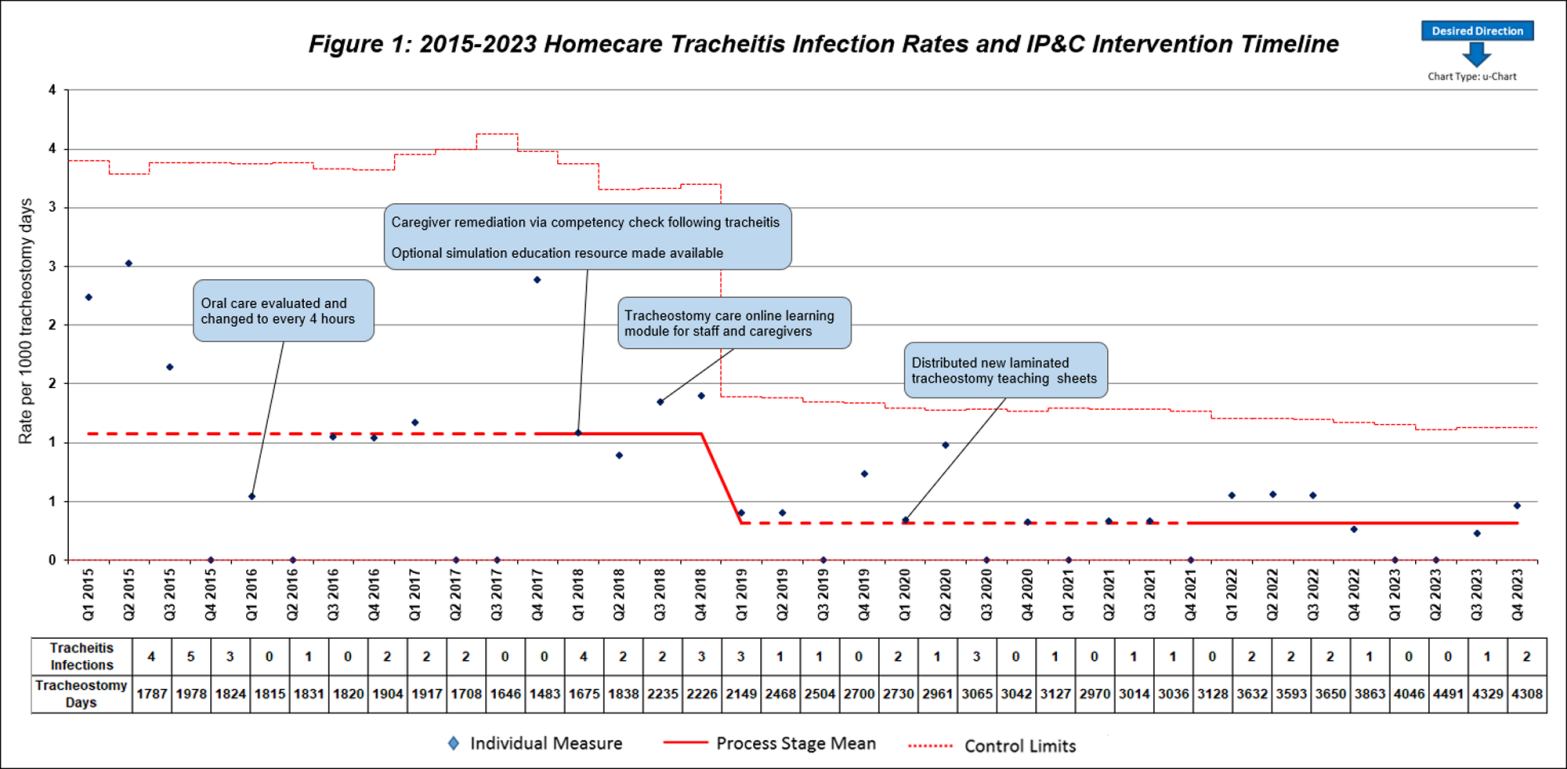

**Figure:**